# N_2_ Generation from Nitric Oxide Coordinated to Iron(III) Porphyrin in Acidic Glycine Buffer

**DOI:** 10.1021/jacs.5c17871

**Published:** 2025-11-24

**Authors:** Atsuki Nakagami, Yoshihito Shiota, Kyosuke Fujikawa, Masahito Kodera, Hiroaki Kitagishi

**Affiliations:** † Department of Molecular Chemistry and Biochemistry, Faculty of Science and Engineering, Doshisha University, Kyotanabe, Kyoto 610-0321, Japan; ‡ Institute for Materials Chemistry and Engineering and IRCCS, 12923 Kyushu University, Fukuoka 819-0395, Japan

## Abstract

Nitric oxide (NO) was efficiently converted to molecular nitrogen (N_2_) in an acidic aqueous solution containing iron­(III) porphyrin encapsulated in the cyclodextrin (CD) nanocavity. The supramolecular iron­(III) porphyrin/CD dimer complexes (hemoCD-P and hemoCD-I), where the iron­(III) is axially coordinated by a nitrogenous ligand (pyridine or imidazole) in the linker of the CD dimer, form stable 6-coordinated ferric nitrosyls {FeNO}^6^ in acidic aqueous solution (pH ∼3). When the solution contained glycine as the buffer component, N_2_ bubbles were significantly generated within several minutes at room temperature. In this system, a new N–N bond is formed on the iron-porphyrin due to the nucleophilic attack of glycine on the {FeNO}^6^ complex. The resulting diazo compound, O=N–NH–CH_2_–COOH ⇄ HO–N=N–CH_2_–COOH, was readily hydrolyzed to generate N_2_ along with a formation of α-hydroxyacid (HO–CH_2_–COOH). The reaction mechanism was evidenced by isotope-labeling experiments using ^15^NO and ^15^N-glycine, quantitative NMR detection of α-hydroxyacid, and theoretical calculation by DFT. The present study will provide the possibility of N–N bond formation promoted by the nucleophilic attack of amines to {FeNO}^6^ on the native heme iron.

## Graphical abstract

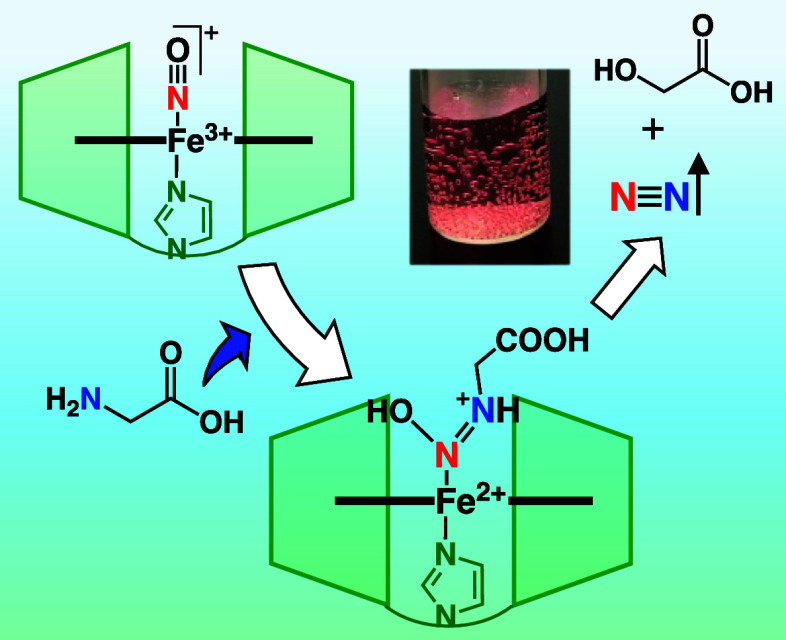

Nitric oxide (NO) coordinates to ferric Fe­(III) and ferrous Fe­(II) porphyrins to form iron-nitrosyl complexes as denoted by {FeNO}^6^ and {FeNO}^7^ electric states in the Enemark–Feltham notation.^1^ The ferric-nitrosyl {FeNO}^6^ complex has one electron delocalized on the Fe–N–O bond that could be attacked by nucleophiles (X) (Figure 1).^1–4^ Various types of Fe-NXO complexes have been investigated, where X = H (hydride), OH (hydroxide), alkane, aryl, and so on.^5–14^ In aqueous solution, ferric nitrosyl complexes are reduced to ferrous ones due to reductive nitrosylation promoted by nucleophilic attack of water and/or hydroxide ions, along with the release of NO_2_^–^.^1,2,5–7^ We have recently reported ferric- and ferrous-nitrosyl complexes of water-soluble 5,10,15,20-tetrakis­(4-sulfonatophenyl)­porphinatoiron (FeTPPS) encapsulated by per-*O*-methylated β-cyclodextrin (CD) dimer linked by a nitrogenous ligand such as imidazole or pyridine (hemoCD-I or hemoCD-P; the chemical structures are shown in Figure S1).^15^ In the ferric nitrosyl complexes, the axial fifth ligand from the CD dimer to FeTPPS in hemoCD-P and -I decreases the Lewis acidity of iron­(III) thus slows down reductive nitrosylation, resulting in the observable ferric nitrosyl complexes obtained at low pH (∼3) at room temperature. These ferric nitrosyl complexes showed a sharp Soret band at 426–427 nm with a bright pink color similar to carbon monoxide (CO) complexes, as the isoelectric state of Fe^III^NO is identical to the CO complex (Fe^II^CO).^16,17^ Structural and binding analyses of the ferric nitrosyl hemoCD complexes were performed in aqueous solution at pH below 7.^15^

**Figure 1:**
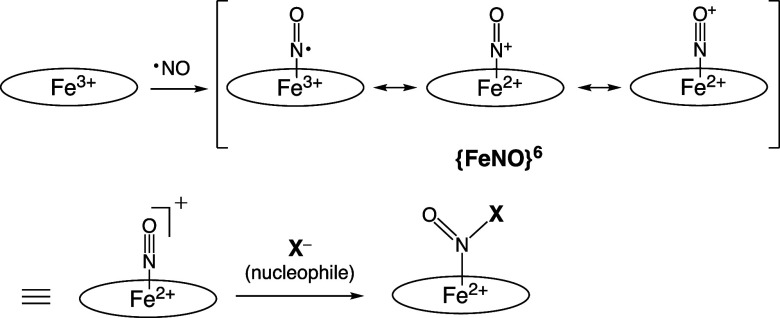
Formation of ferric nitrosyl {FeNO}^6^ porphyrin complex followed by the reaction with nucleophile.

During the experiments on hemoCD, we unexpectedly observed gas bubble generation in the solution containing ferric nitrosyl complexes within several minutes (Figure 2a). Significant amounts of insoluble gas appeared as bubbles when NO was introduced into the solution of ferric hemoCD-P and -I in glycine-HCl buffer at pH 3. In control group containing Fe­(III)­TPPS, free base TPPS, or Fe^III^TPPS complexed with two 2,3,6-tri-*O*-methyl-β-CD (TMe-β-CD, an analogue of hemoCD without an axial ligand), there were little gas bubbles generated under the same conditions (Figure S2). We previously confirmed that Fe^III^TPPS without the CD dimers does not form stable ferric nitrosyl complexes in water because of rapid reductive nitrosylation.^15^ Therefore, the formation of stable 6-coordinated ferric nitrosyl complexes would be crucial for the gas generation. The headspace gas chromatography (GC) analysis identified that the bubbles contained significant amount of nitrogen (N_2_) (Figure 2b). The water-solubility of N_2_ is significantly low (∼0.7 mM) compared to NO (∼1.9 mM) or other related gases (N_2_O, ∼24 mM; O_2_, ∼1.3 mM) at room temperature (25 °C).^18^ Other gas components were not detected in the GC chart. This reaction was observed under air; the solution thus contained 80% saturated N_2_ prior to the reaction. The newly generated N_2_ should mostly escape from the solution as bubbles. The amount of N_2_ (μmol) was quantified by the water displacement method, which rapidly increased and reached ∼30 μmol at 60 min when NO gas (30 cc) was added to the solution containing hemoCD-I (25 μM in 1.5 mL, i.e. 38 nmol) (Figure 2c-e). Interestingly, N_2_ was not detected when HCl_aq_ without glycine was used for adjusting the pH, suggesting that NO, ferric hemoCD, and glycine were involved in N_2_ generation.

**Figure 2:**
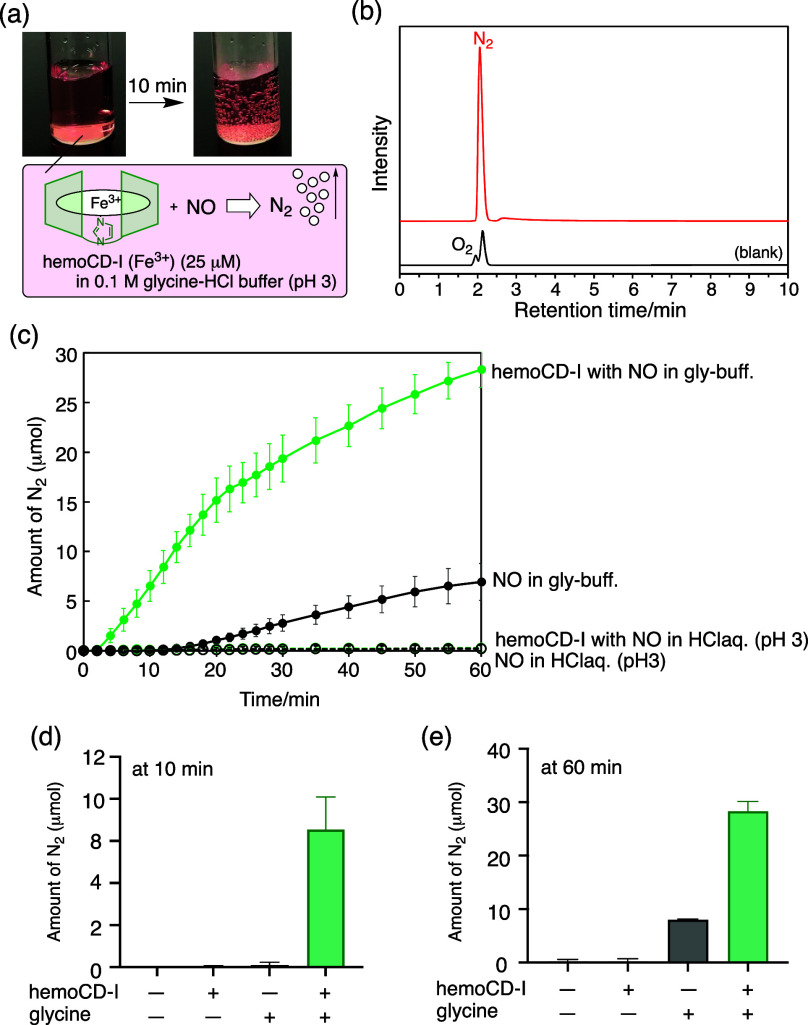
(a) Formation of N_2_ bubbles from the aqueous solution containing hemoCD-I and NO in 0.1 M glycine-HCl buffer at pH 3. (b) Gas chromatograms for the headspace sample in the NO-hemoCD-I complex solution. (c-e) Quantitative results for the N_2_ generation from NO at pH 3 in the absence and presence of hemoCD-I and glycine.

The mass number of N_2_ generated was measured by GC-MS (Figure 3). When ^15^NO was applied to the hemoCD-I solution, the mass ion peak was found at 29 *m*/*z* due to hemilabeled N_2_ gas (^15^N^14^N) (Figure 3b). Using ^15^N-labeled glycine (H_2_^15^N–CH_2_–COOH) as the buffer component, we found the peaks at 29 and 30 from the ^14^NO and ^15^NO applied solutions, respectively, due to ^14^N^15^N and ^15^N_2_ formation (Figure 3c,d). These results clearly support the hypothesis that glycine should be involved in N_2_ generation from the ferric nitrosyl hemoCD complex.

**Figure 3:**
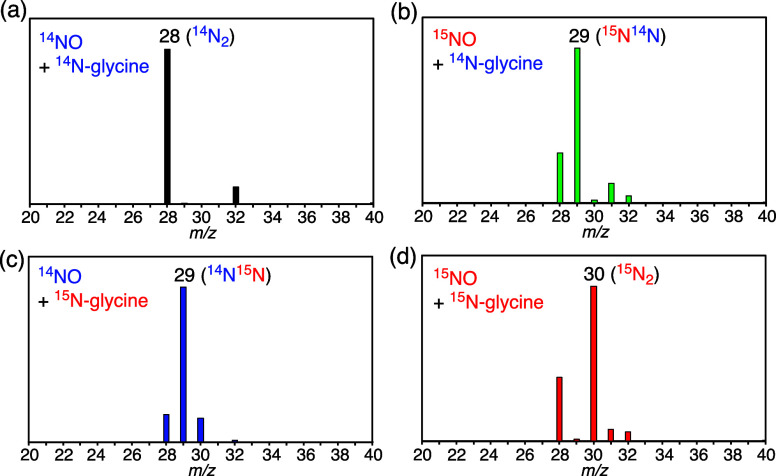
Mass spectra of N_2_ generated from the aqueous NO-hemoCD-I solution in 0.1 M glycine-HCl buffer at pH 3 under (a) ^14^NO with ^14^N-glycine, (b) ^15^NO with ^14^N-glycine, (c) ^14^NO with ^15^N-glycine, and (d) ^15^NO with ^15^N-glycine.

We propose the mechanism of N_2_ generation (Scheme 1). Once the {FeNO}^6^ complex of hemoCD (NO-hemoCD-I) formed in acidic glycine buffer, nucleophilic attack of glycine on the complex resulted in the N–N bond formation. Dissociation of the diazo compound from Fe^2+^ followed by proton-transfer and hydrolysis releases N_2_ with α-hydroxyacid as the products. The ferrous porphyrin is transiently formed and autoxidized to a ferric state. This was evidenced by the control experiment with CO (); the amount of N_2_ generated significantly decreased because autoxidation from ferrous to ferric hemoCD was suppressed in the presence of CO.^19^ As reported previously, O_2_-adducts of hemoCD-P and hemoCD-I were relatively stable at neutral pH with a half lifetime of several hours in water at neutral pHs, whereas in acidic solution the autoxidation was significantly accelerated and completed within 1 min.^20,21^ The ferrous hemoCD could also bind to NO.^15^ However, ferrous nitrosyl hemoCD-I was found to be quite unstable (not detected at pH 3) and rapidly oxidized to the ferric state in acidic solution (Figure S4). Therefore, rapid autoxidation of ferrous hemoCD-I enables the catalytic cycle of N_2_ formation in the presence of excess glycine.

**Scheme 1:**
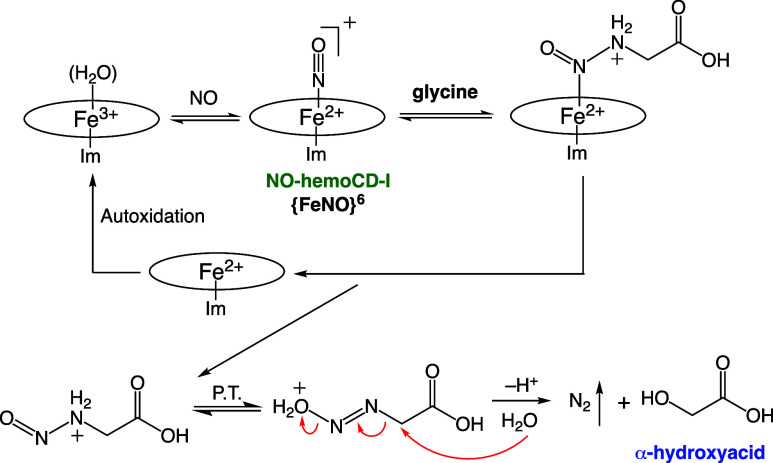
Plausible Mechanism for N_2_ Generation from NO Bound to hemoCD-I Involving Glycine in the Acidic Buffer

To confirm the proposed mechanism, α-hydroxyacid was detected and quantified using NMR. For NMR analysis, NO gas (30 cc) was introduced into the solution (1.5 mL) containing ferric hemoCD-I (25 μM) in 0.1 M glycine-HCl buffer. After gas generation ceased, the solution was once freeze-dried and then the residue was redissolved in D_2_O. (Figure 4a). ^1^H (Figure 4b) and ^13^C NMR spectra (Figure 4c) clearly detected the peaks at 4.19 ppm (^1^H), 60.0 and 177.1 ppm (^13^C), which are assignable to α-hydroxyacid^22^ in addition to the original peaks due to glycine (3.66 ppm for ^1^H, 41.6 and 172.2 ppm for ^13^C). From the integral value of the signals in the ^1^H NMR spectrum (Figure S5), α-hydroxyacid was generated at 32 ± 2 μmol for 60 min reaction time, which was quite consistent with the amount of N_2_ quantified under the same condition (see Figure 2c,e). The quantitative formation of α-hydroxyacid with N_2_ in this system strongly supports the proposed mechanism.

**Figure 4:**
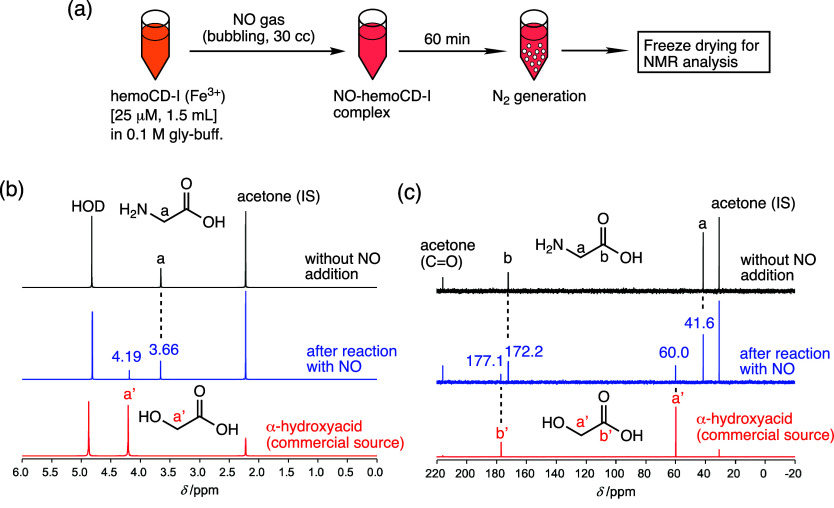
Detection and quantification of α-hydroxyacid. (a) Experimental procedure. ^1^H (b) and ^13^C NMR spectra (c) of the residual samples without (upper, black lines) and with NO addition (middle, blue lines) to the hemoCD-I solution (25 μM, 1.5 mL) in 0.1 M glycine-HCl buffer at pH 3. The bottom spectra in (b) and (c) (red lines) were obtained from an authentic sample (α-hydroxyacid). Quantitative analysis based on the integral values is shown in the Supporting Information (Figure S5).

To theoretically support the mechanism, potential energy surfaces of the reaction intermediates and these possible transition states were calculated by DFT at the B3LYP level of theory. A simplified 5,10,15,20-tetraphenylporphinatoiron­(III) with an *N*-methylimidazole as the axial fifth ligand was used as the model structure. Figure 5 shows potential energy diagrams of N–N bond formation and diazo compound dissociation reactions. For the transformation from the ferric-nitrosyl complex (**RC**) to its glycine adduct (**Int**), proton transfer (PT) should be involved from NH_2_ (glycine) to the oxygen atom of NO. The *cis* form (*cis* **Int**) at the N=N bond is relatively stable compared to the *trans* one (*trans* **Int**). The relative energy of the transition state (**TS**) for N–N bond formation was too high to proceed at room temperature (46.6 kcal/mol). Interestingly, the activation energy was significantly lowered to 28.1 kcal/mol when a water molecule was involved in the PT process, which was possible by forming a water-bridged structure between coordinated NO and glycine. Therefore, the transition state with a water molecule (**TSw**) is plausible as illustrated in Figure 5 (water-assisted N–N bond formation). From the intermediate, the Fe–N bond tends to dissociate to ferric and diazo radical (6.9 kcal/mol) rather than the ferrous state with diazo cation (47.8 kcal/mol). Once the radical species, HO–N^•^–NH–CH_2_–COOH, is released from the iron-porphyrin, N_2_ is liberated by following three steps (the DFT calculation is summarized in Figure S6); (1) dehydration from HO–N^•^–NH–CH_2_–COOH to ^•^N=N–CH_2_–COOH (−34.2 kcal/mol), (2) one electron oxidation from ^•^N=N–CH_2_–COOH to N^+^=N–CH_2_–COOH, and (3) a S_N_2-type reaction of N^+^=N–CH_2_–COOH with H_2_O to give α-hydroxyacid and N_2_ (−60.3 kcal/mol). As for the oxidation (step 2), ferric hemoCD is thought to function as an electron acceptor. As confirmed by the experiment with CO, the ferrous state was involved during the catalytic process. Integrating these experimental and theoretical data, we propose the concerted mechanism for the N_2_ release that could occur in the molecular cage of methylated CD dimer as shown in Figure 5 (N_2_ release from diazo radical in the molecular cage). The unfavorable process of the ferrous state formation with the diazo cation would be compensated by the exothermic release of N_2_.

**Figure 5:**
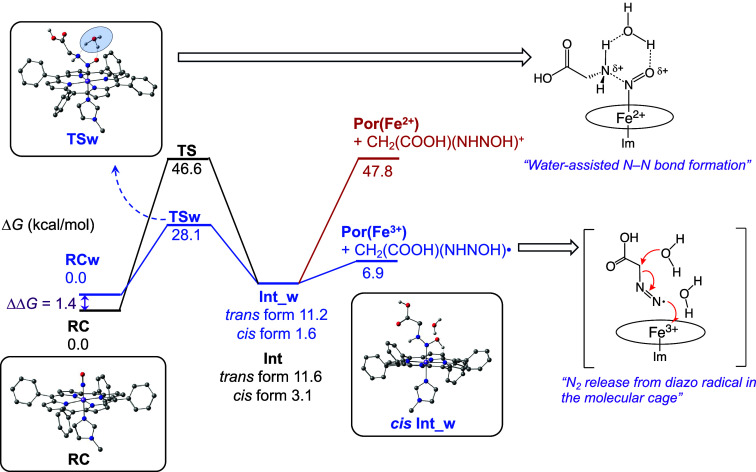
Potential energy diagrams for N–N bond formation of ferric nitrosyl {FeNO}^6^ porphyrin complex with glycine, followed by dissociation of diazo compound: **RC**, reaction complex; **TS**, transition state; **Int**, intermediate. The “**w**” means the state with a proximal water molecule. The energy diagrams for decomposition of the diazo compound to release N_2_ are shown in the Supporting Information (Figure S6).

In the control solution without hemoCD (NO in glycine-HCl buffer), a significant amount of N_2_ was detected after a 10–15 min induction period (see Figure 2c and e). This could be explained by the reaction of HNO_2_ with glycine (Figure S7).^23^ In acidic solution, HNO_2_ formed due to disproportionation (4NO + H_2_O → N_2_O + 2HNO_2_),^24^ showing characteristic absorption at 300–400 nm (Figure S8).^25^ The absorbance gradually disappeared regardless of the presence of hemoCD (Figure S8). Therefore, N_2_ generation in the hemoCD system proceeds through a pathway independent of the NO disproportionation. We then tested the N_2_ generation reaction with alanine instead of glycine (Figure S9). Interestingly, N_2_ was not detected beyond the blank system. Considering the specific structure of hemoCD (Figure S10), the narrow cleft between two methylated CD ions sterically hindered the methyl group of alanine. Therefore, glycine is the suitable nucleophile to the Fe^III^NO complex buried in the deep cleft of hemoCD-I.

Direct conversion from NO to N_2_ is performed in an industrial process (denitrification). In the industry, ammonia is widely used as a reductant in the presence of metal-based catalysts.^26–28^ Although the industrial process is typically conducted at high temperature (200–300 °C), the reaction mechanism is considered basically the same that observed in this study; i.e., nucleophilic attack of ammonia to NO bound on the metal catalysts to promote the N–N bond formation. The metal-nitrosyl chemistry including the N–N bond formation with N-nucleophiles has a long history.^29,30^ To the best of our knowledge, however, the present hemoCD system is the first of direct conversion from NO to N_2_ occurring on the heme iron. In the NO reduction system by native heme proteins such as NO reductase (NOR), two NO molecules are typically involved for the N–N bond formation and the product (N_2_O) is further reduced by another enzyme (N_2_O reductase).^31–37^ While the reactions of water-soluble porphyrins with NO have been well studied at various pHs, N_2_ generation has not been identified so far.^35,38–40^ This is probably due to the lack of stably coordinated axial fifth ligand that can stabilize ferric NO ({FeNO}^6^) complex under acidic conditions. In this study, in addition to a stable 6-coordinated NO complex formation in the hemoCD system, the use of glycine as a buffer component enabled us to observe the unexpected reaction from NO to N_2_. Although this reaction proceeded under specific conditions, we believe that the present insight from the biomimetic model study provides a new possibility to consider the NO reduction process in a native nitrogen cycle.

## Supplementary Material







## Notes

Experimental section (materials, spectroscopic methods, GC analysis, NMR measurements, DFT calculations), full chemical structures of hemoCDs, a series of control experiments, a quantitative NMR analysis for α-hydroxyacid, DFT calculations for diazo compounds, a reaction path for HNO_2_ with glycine, and the molecular model of NO-hemoCD-I with glycine/alanine (PDF)
Time lapse movie of N_2_ generation with control samples (MP4)
Cartesian coordinates for all compounds (XYZ)
