# Supplementary material for: N2 Generation from Nitric Oxide Coordinated to Iron(III) Porphyrin in Acidic Glycine Buffer
Source: J Am Chem Soc. 2025 Nov 24;147(52):47898–903. doi: 10.1021/jacs.5c17871 (PMC12766681; doi:10.1021/jacs.5c17871)
Supplement: Supplementary file 1 [file ja5c17871_si_001.pdf]

Supporting Information for:

N_2 Generation from Nitric Oxide Coordinated to Iron(III)porphyrin in Acidic Glycine Buffer

Atsuki Nakagami,[1] Yoshihito Shiota,[2] Kyosuke Fujikawa,[1] Masahito Kodera,[1] and Hiroaki Kitagishi*[1]

[1]Department of Molecular Chemistry and Biochemistry, Faculty of Science and Engineering, Doshisha University, Kyotanabe, Kyoto 610-0321, Japan

[2]Institute for Materials Chemistry and Engineering and IRCCS, Kyushu University, Fukuoka 819-0395, Japan

Experimental Section

Materials. The cyclodextrin dimer (Im3CD) and FeTPPS were prepared as described previously.[S1] NO gas was purchased from Taiyo Nippon Sanso and used with passing through 5 M NaOH solution. ^{15}NO gas (^{15}N ≧ 99.9Atom%) was purchased from Shoko Science. ^{15}N-glycine was purchased from MedChemExpress (Product ID: HY-Y0966S). All other reagents were purchased and used as received. The aqueous solutions of 0.1 M glycine and 0.1 M HCl were used to prepare the glycine buffer at pH 3.

Spectroscopic measurements.

UV-vis spectra were recorded on a Shimadzu UV-2600i spectrophotometer with a thermostatic cell holder. ^{1}H and ^{13}C NMR spectra were recorded at ambient temperature on a JEOL JNM-ECA 500 spectrometer, operating at 500 MHz for ^{1}H NMR and 125 MHz for ^{13}C NMR. Chemical shifts (δ) are given in ppm relative to acetone as an internal standard (2.22 ppm and 30.89 ppm ($(\underline{C}H_3)_2CO$) in D_2O for ^{1}H and ^{13}C, respectively[S2]).

Gas chromatography (GC) and gas chromatography-mass spectrometry (GC-MS)

NO gas (30 cc) was bubbled into the solution of 1.5 mL of hemoCD-I (25 μM) in 0.1 M glycine-HCl buffer at pH 3 at 25°C. The produced gas was collected by the water displacement method. After 1 hour, the gas phase was collected by a gas tight syringe and analyzed by gas chromatography (GC). The GC conditions were as follows: detector = TCD; column = SHINCARBON ST 50/80 (3.0 mm, 2.0 m); carrier gas = He; column temperature = 40°C; temperature at vaporizing chamber and detector = 120°C; flow rate = 50 ml/min. For GC/MS, a portion of the generated gas was injected by a micro syringe (5 μL) and analyzed. The GC/MS conditions were as follows: column = InertCap 1701 capillary column (0.25 mm, 60 m); carrier gas = He; column temperature = 40°C; temperature at vaporizing chamber = 120°C; flow rate = 0.89 mL/min; temperature at the ion source and the interface = 200°C.

Quantification of produced gas.

The solution of hemoCD-I (25 μM, 1.5 mL) in 0.1 M glycine-HCl buffer at pH 3 was prepared in a 1.5 mL glass vial at 25°C. Then, NO gas (30 cc) was bubbled into the

solution. The head space was quickly replaced by helium, and the vial was sealed with a septum screw cap. A needle that connected silicon tube (ϕ1.5 mm) was inserted to the vial. The produced gas was quantified by the water displacement method.

Quantification of α-hydroxyacid by NMR spectroscopy.

The amount of α-hydroxyacid was quantified by NMR spectroscopy. The same solution which was used for quantification of produced gas was used for this analysis. After gas bubble generation ceased, the solution was once freeze-dried. Then, an aliquot of the residue (~ 5 mg) was re-dissolved in D_2O (0.6 mL). Acetone (5 μL) was added as the internal standard. The amount of α-hydroxyacid was determined as follows; the ratio of the integral values between glycine (δ = 3.66 ppm) and α-hydroxyacid (δ = 4.19 ppm) was obtained in the ^{1}H NMR spectrum, then the amount of α-hydroxyacid was calculated based on the initial amount of glycine (1.5 mmol in 1.5 mL of 0.1 M glycine solution) and the ratio of glycine/α-hydroxyacid (see Figure S5 for example).

DFT calculations.

All full-optimized structures were obtained by using the B3LYP functional[S3,S4] as implemented in the Gaussian 16 packages.[S5] The Wachter-Hay basis set[S6,S7] was used for the Fe atom, and the D95** basis set[S8] was used for the C, N, O, and H atoms. By calculating the analytical harmonic vibration frequencies, we confirmed that the obtained local minima have no imaginary frequency. The contribution of free energy corrections at 298.15 K was included to predict reliable energetics. As a model for the hydrophobic field, calculations were performed under vacuum conditions without considering the solvent effect.

References

S1) Mao, Q.; Zhao, X.; Kiriyama, A.; Negi, S.; Fukuda, Y.; Yoshioka, H.; Kawaguchi, A. T.; Motterlini, R.; Foresti, R.; Kitagishi, H. A Synthetic porphyrin as an effective dual antidote against carbon monoxide and cyanide poisoning. *Proc. Natl. Acad. Sci. U.S.A.* **2023**, *120*, e2209924120.

S2) Babij, N. R.; McCusker, E. O.; Whiteker, G. T.; Canturk, B.; Choy, N.; Creemer, L. C.; De Amicis, C. V.; Hewlett, N. M.; Johnson, P. L.; Knobelsdorf, J. A.; Li, E.;

Lorsbach, B. A.; Nugent, B. M.; Ryan, S. J.; Smith, M. R.; Yang, Q. NMR Chemical Shifts of Trace Impurities: Industrially Preferred Solvents Used in Process and Green Chemistry. *Org. Process. Res. Dev.* **2016**, *20*, 661–667.
S3) Becke, A. D. Density-functional thermochemistry. III. The role of exact exchange. *J. Chem. Phys.*, **1993**, *98*, 5648–5652.
S4) Lee, C.; Yang, W.; Parr, R. G. Development of the Colle-Salvetti correlation-energy formula into a functional of the electron density. *Phys. Rev. B,* **1988**, *37*, 785–789.
S5) Gaussian 16, Revision C.01, Frisch, M. J.; Trucks, G. W.; Schlegel, H. B.; Scuseria, G. E.; Robb, M. A.; Cheeseman, J. R.; Scalmani, G.; Barone, V.; Petersson, G. A.; Nakatsuji, H.; Li, X.; Caricato, M.; Marenich, A. V.; Bloino, J.; Janesko, B. G.; Gomperts, R.; Mennucci, B.; Hratchian, H. P.; Ortiz, J. V.; Izmaylov, A. F.; Sonnenberg, J. L.; Williams-Young, D.; Ding, F.; Lipparini, F.; Egidi, F.; Goings, J.; Peng, B.; Petrone, A.; Henderson, T.; Ranasinghe, D.; Zakrzewski, V. G.; Gao, J.; Rega, N.; Zheng, G.; Liang, W.; Hada, M.; Ehara, M.; Toyota, K.; Fukuda, R.; Hasegawa, J.; Ishida, M.; Nakajima, T.; Honda, Y.; Kitao, O.; Nakai, H.; Vreven, T.; Throssell, K.; Montgomery, Jr., J. A.; Peralta, J. E.; Ogliaro, F.; Bearpark, M. J.; Heyd, J. J.; Brothers, E. N.; Kudin, K. N.; Staroverov, V. N.; Keith, T. A.; Kobayashi, R.; Normand, J.; Raghavachari, K.; Rendell, A. P.; Burant, J. C.; yengar, S. S. I.; Tomasi, J.; Cossi, M.; Millam, J. M.; Klene, M.; Adamo, C.; Cammi, R.; Ochterski, J. W.; Martin, R. L.; Morokuma, K.; Farkas, O.; Foresman, J. B.; Fox, D. J. Gaussian, Inc., Wallingford CT, 2016.
S6) Wachters, A. J. H. Gaussian Basis Set for Molecular Wavefunctions Containing Third-Row Atoms. *J. Chem. Phys.* **1970**, *52,* 1033–1036.
S7) Hay, P. J. Gaussian basis sets for molecular calculations. The representation of 3*d* orbitals in transition-metal atoms. *J. Chem. Phys.*, **1977,** *66*, 4377–4384.
S8) Dunning Jr., T. H.; Hay, P. J. *in Modern Theoretical Chemistry*, Schaefer III, H. F., Eds.; Vol. 3; Plenum, 1977, pp 1–28.
S9) Tejero, J.; Santolini, J.; Stuehr, D. J. Fast ferrous heme–NO oxidation in nitric oxide synthase. *FEBS J.* **2009**, *276*, 4505–4514.

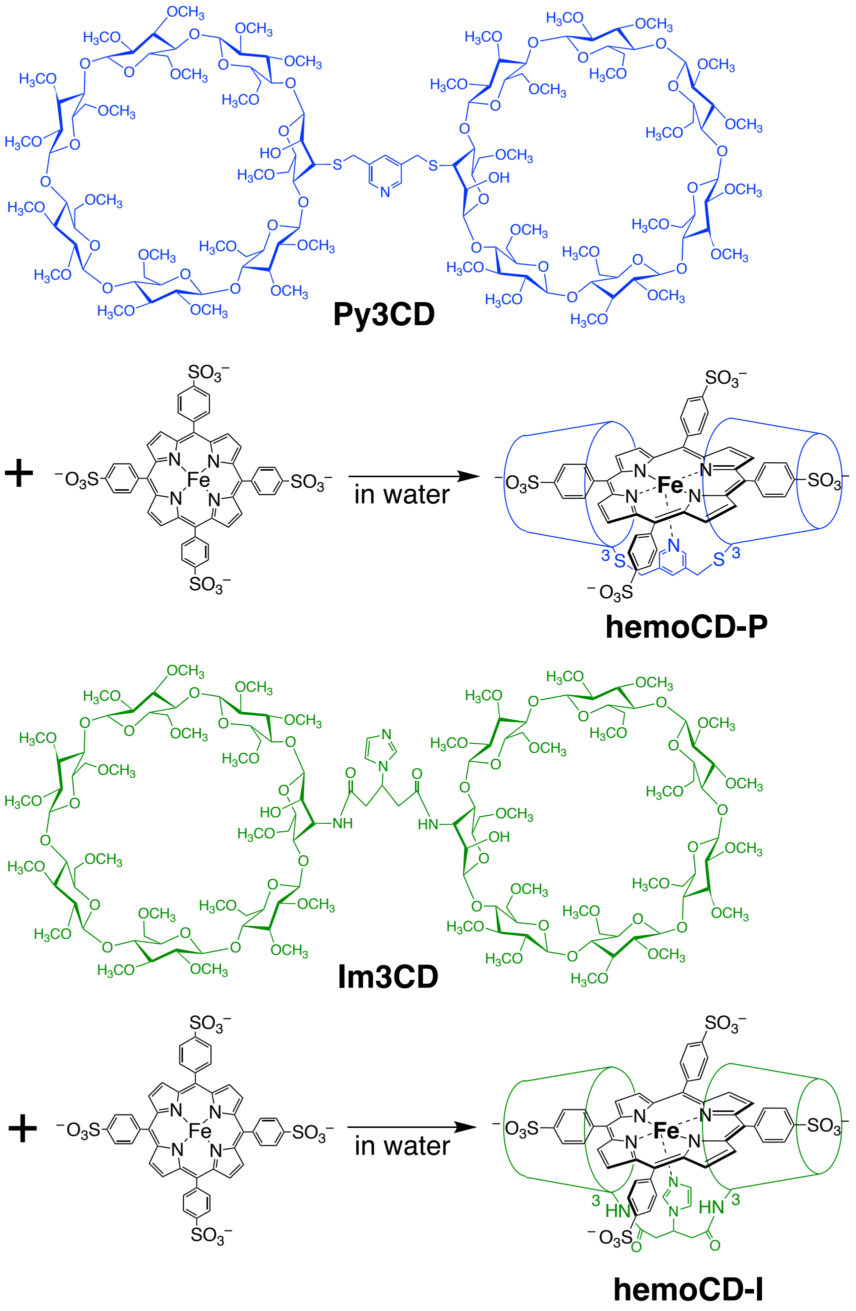

Figure S1. The chemical structures of the CD dimers (Py3CD, Im3CD) and their complexes with Fe(III)TPPS in water.

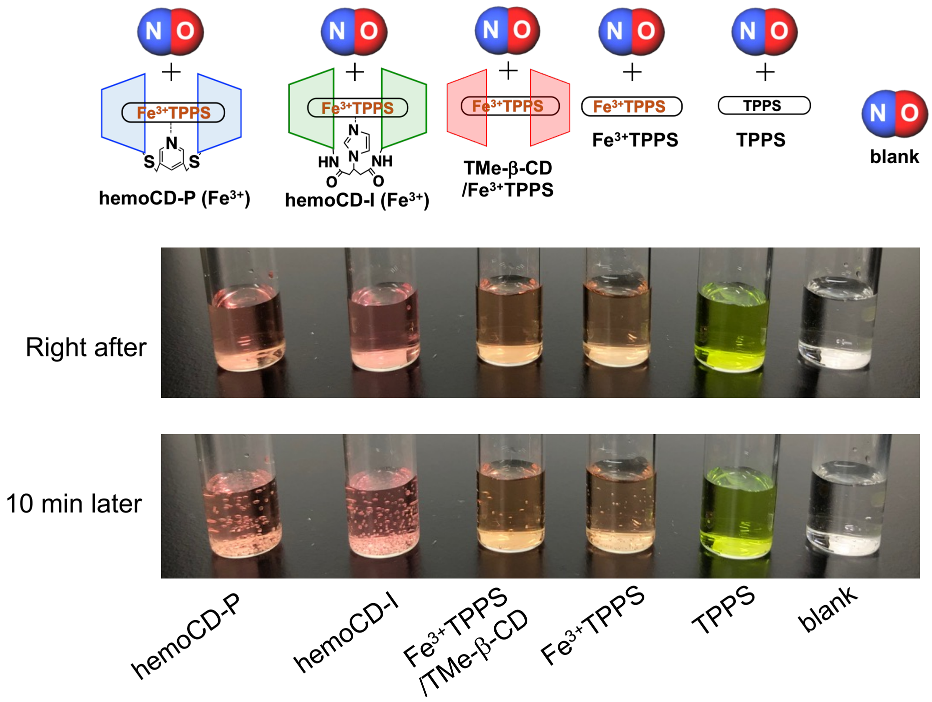

Figure S2. Visual observation of the solutions upon addition of NO gas in 0.1 M glycine-HCl buffer at pH 3 and 25°C. From the left, solution contains hemoCD-P (25 μM), hemoCD-I (25 μM), TMe-β-CD/Fe^{3+}TPPS (60 μM/25 μM), Fe^{3+}TPPS (25 μM), TPPS (25 μM), respectively. The significant gas generation was observed in samples contains hemoCD-P and hemoCD-I in NO dissolved acidic solution after 10 min incubation time. The timelapse movie of this observation is also available at a supporting information file in the MPEG-4 format.

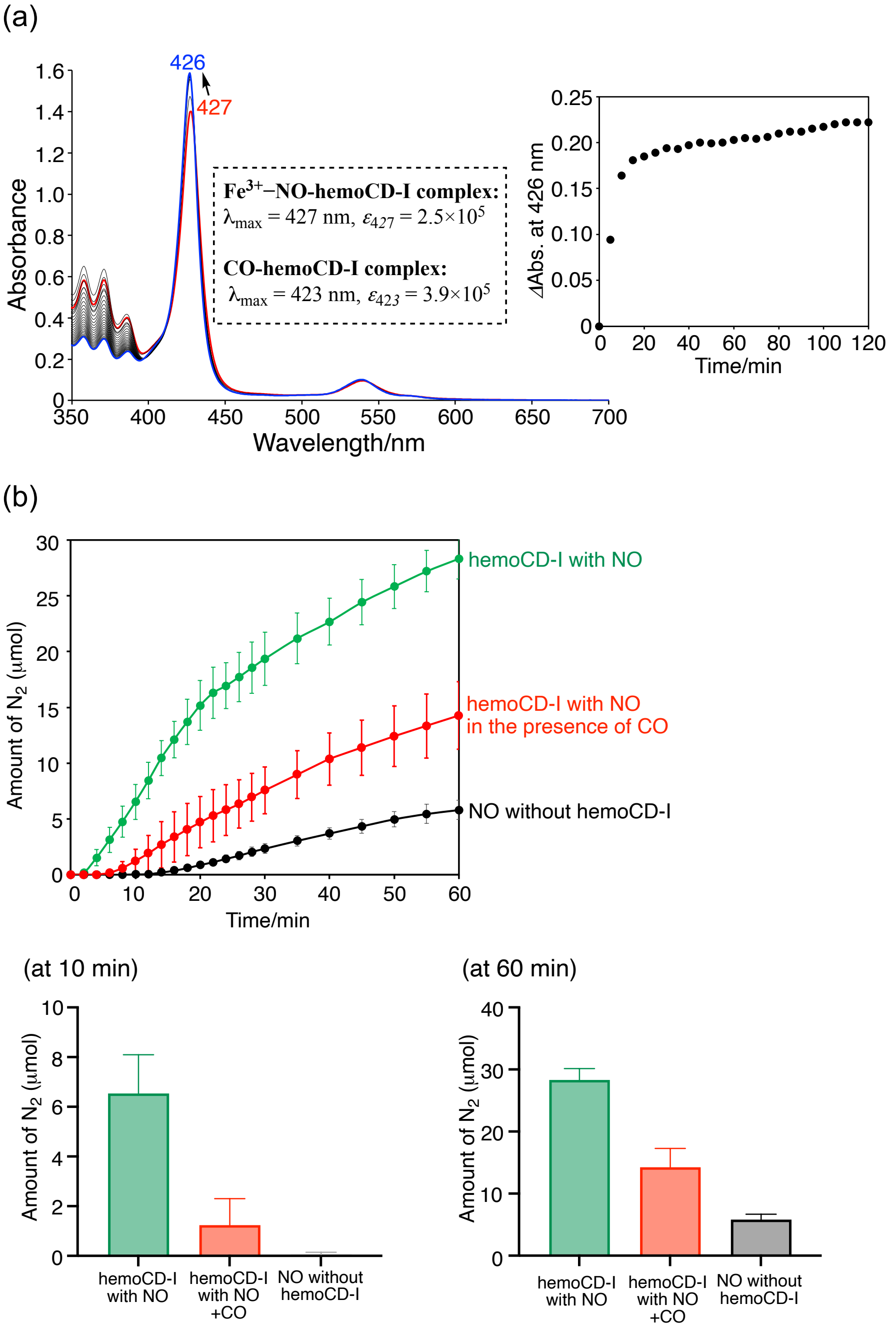

Figure S3. N_2 gas generation from NO with hemoCD-I in 0.1 M glycine-HCl buffer at pH 3 and at 25°C in the presence of carbon monoxide (CO). The UV-vis spectral change of hemoCD-I with NO in the presence of CO (a). The quantitative results for N_2 generation from NO in the absence and presence of hemoCD-I or CO (b). In both experiment, CO gas was bubbled into the glycine-HCl buffer for 5 min prior to NO gas bubbling. The blue shift and the increase in absorbance suggested that the CO-bound hemoCD-I was formed during the reaction. In addition, N_2 production was partly suppressed by CO, indicating that the ferrous state of hemoCD-I should be involved in the reaction cycle.

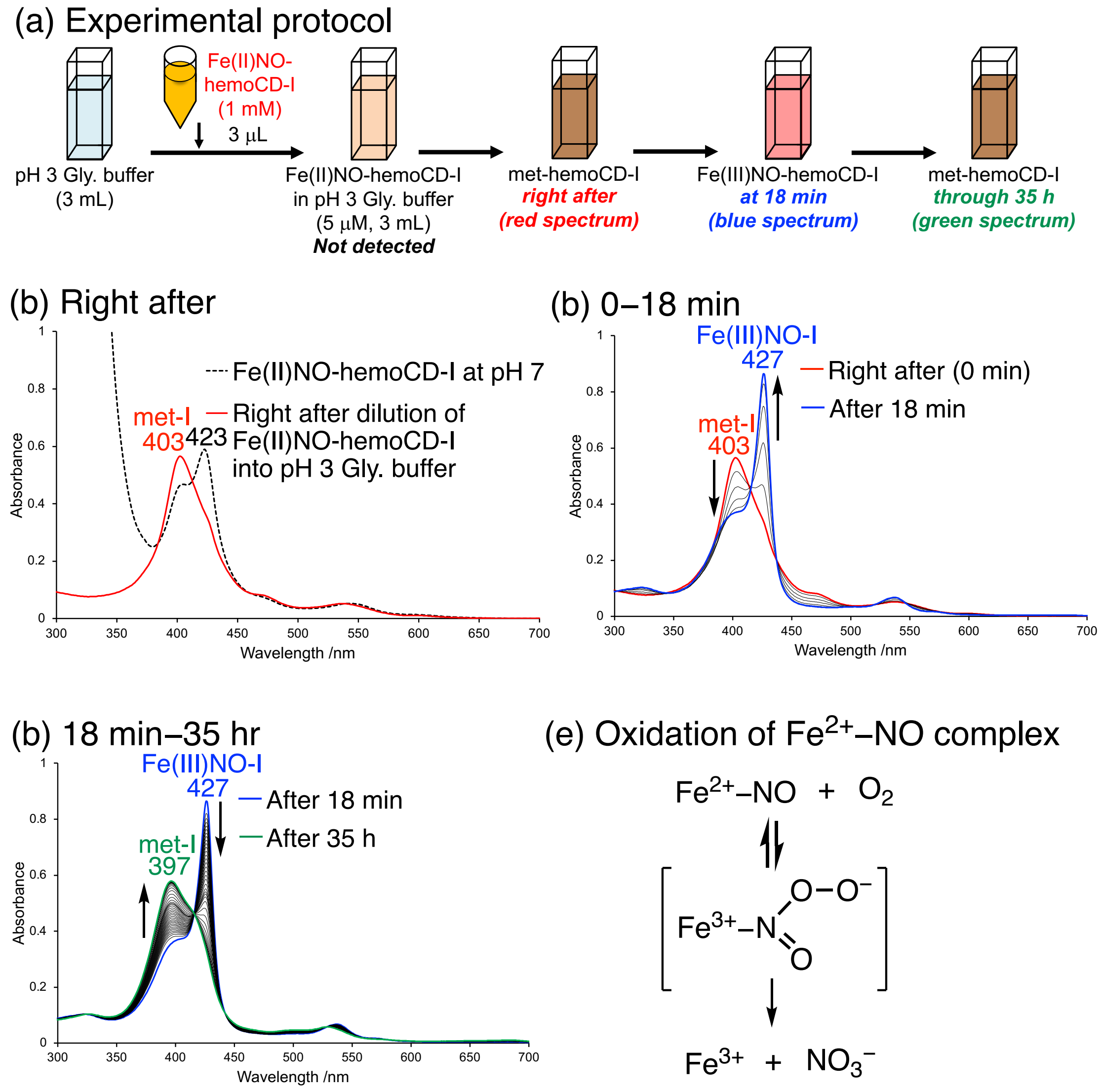

Figure S4. UV-vis spectral changes of ferrous nitrosyl hemoCD-I in 0.1M glycine-HCl buffer at pH 3 and 25°C. Ferrous nitrosyl hemoCD-I was prepared in advance as follows; excess $Na_2S_2O_4$ was added to hemoCD-I (1.0 mM), then NO gas bubbled for 30 sec in 0.1 M phosphate buffer at pH 7 to form NO-Fe^{2+}-hemoCD-I. A proportion of the solution (3 μL) was diluted into 0.1 M glycine-HCl buffer (3 mL) at pH 3 (a). Observed spectral changes were consisted with three steps. In first step (right after dilution), observed spectrum (shown in red) was identical to ferric hemoCD-I. NO-Fe^{2+}-hemoCD-I was not detected at pH3 because of rapid oxidation (shown in black dot for comparison) (b). In a second step (up to 18 min), the formation of NO-Fe^{3+}−hemoCD-I was confirmed, which suggested that the residual NO bound to Fe^{3+}-hemoCD-I (c). In third step (from 18 min to 35 h), the gradual change from NO-Fe^{3+}-hemoCD-I to Fe^{3+}-hemoCD-I was observed (d). (e) The proposed mechanism for the oxidation of ferrous nitrosyl complex.[S9]

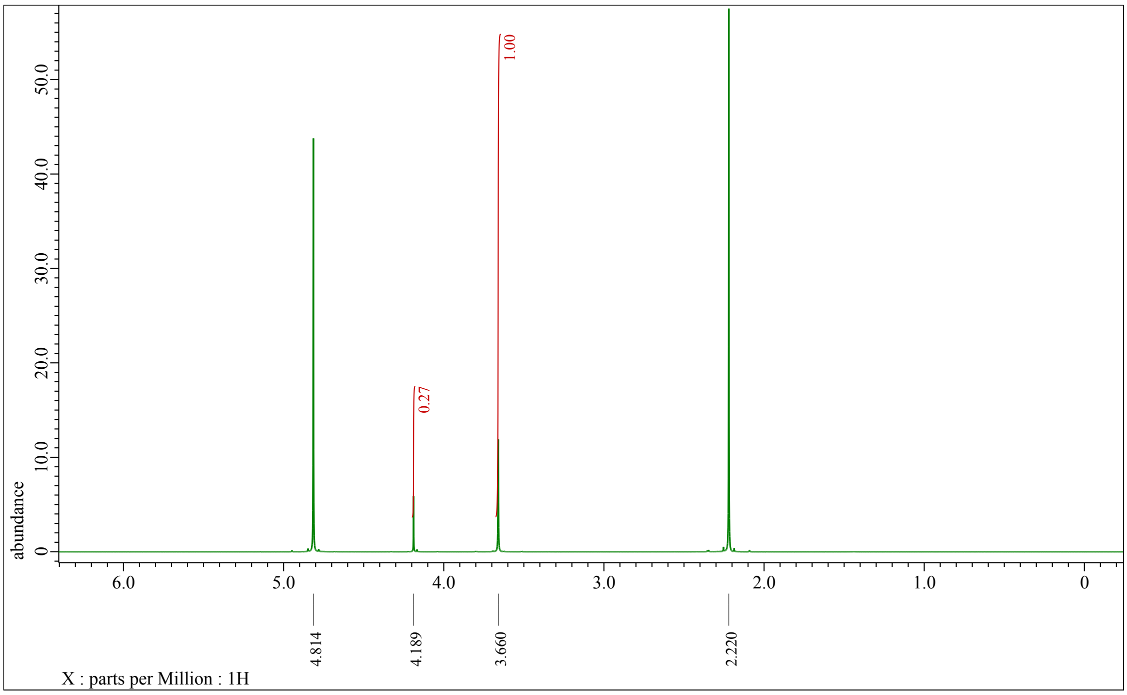

Figure S5. ^{1}H NMR spectrum with integral values of the residual sample in the solution after the reaction between met-hemoCD-I and NO in 0.1 M glycine-HCl buffer at pH 3. The amount of α-hydroxyacid was quantified by the ratio of the integral values between glycine (δ = 3.66 ppm) and α-hydroxyacid (δ = 4.19 ppm). In this case, [glycine] : [α-hydroxyacid] = 1.0 : 0.27. Since the initial amount of glycine is 0.15 mmol (1.5 mL of 0.1 M glycine sol.), the amount of α-hydroxyacid was determined to be 31.9 μmol (0.15 mmol × 0.27/(1 + 0.27)).

<First step: dehydration>

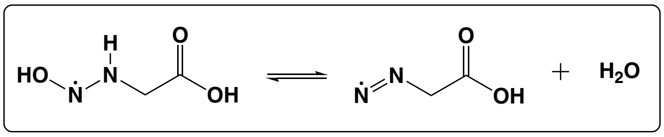

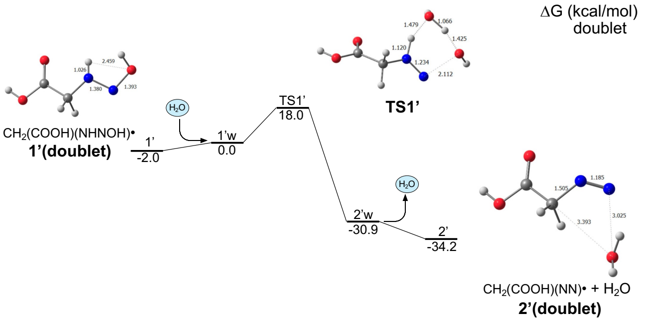

<Second step: an electron transfer>

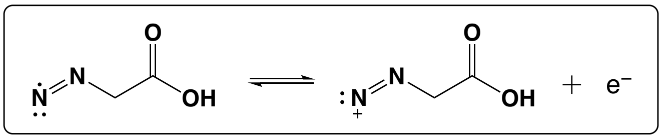

<Third step: S_N2 reaction by a water molecule>

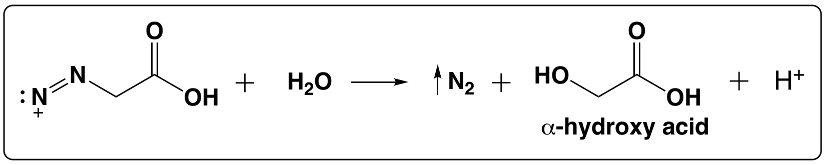

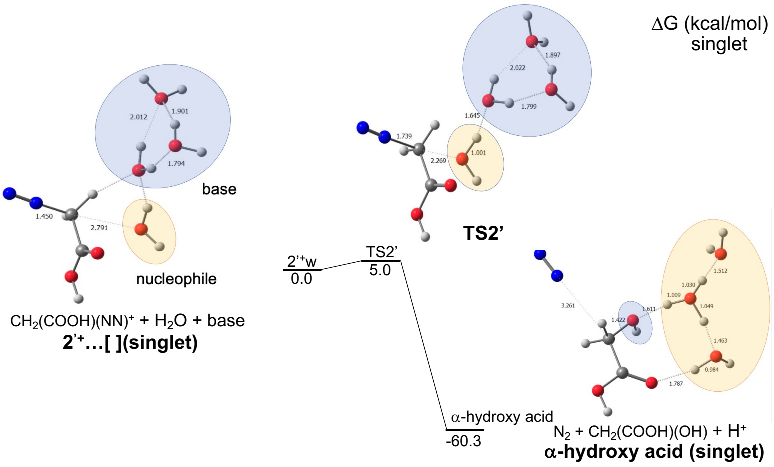

Figure S6. Relative energy diagrams for N_2 production from a diazo radical compound (HO-N(•)-NH-CH_2-COOH). Dehydration reaction occurs first, followed by one electron oxidation. At the third step, N_2 and α-hydroxyacid are produced by S_N2-type reaction by water molecule, where a water-cluster, shown in the blue circle, is expected to form and work as the base to promote the nucleophilic attack. As a solution model, solvent effect of water was included using the corresponding polarizable continuum model (PCM).

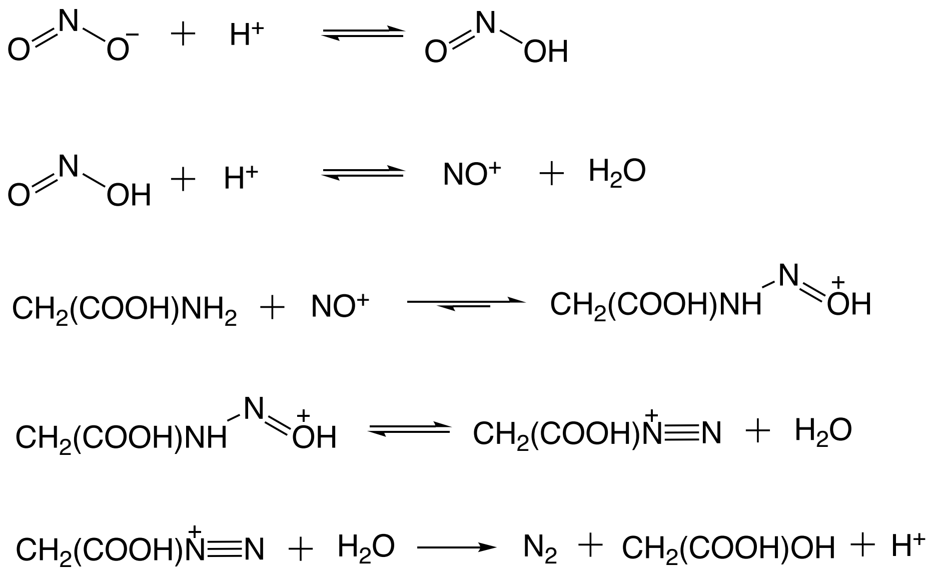

Figure S7. Proposed reaction path for N_2 production from HNO_2 with glycine in blank system (without hemoCD). In this system, NO gas was introduced into 0.1 M glycine-HCl buffer solution. In acidic condition, a portion of NO can be converted to HNO_2 in acidic buffer because NO has a high redox potential in acidic condition [Latimer Diagram of Nitrogen at pH 0, see Lehnert et al., *Chem. Rev.* **2021**, 121, 14682–14905], and it easily cause disproportionation as mentioned in the main text. Subsequently, produced HNO_2 generates nitrosyl cation (NO^+). NO^+ readily reacts with glycine to give a *N*-nitroso amine compound. Subsequently, the *N*-nitroso amine compound is dehydrated, then generate N_2 and α-hydroxyacid. This reaction occurs but proceeds more slowly than the system with hemoCD.

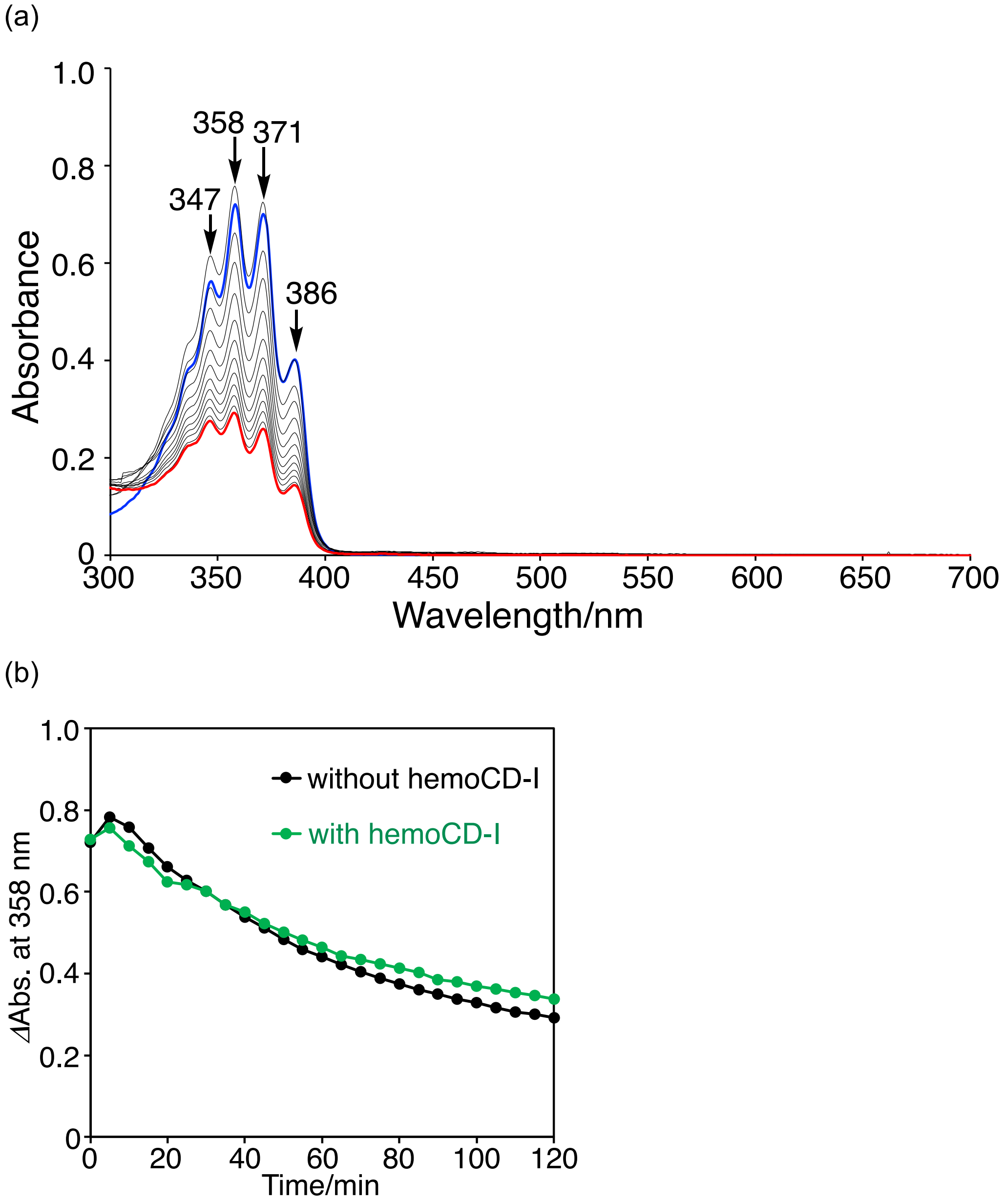

Figure S8. (a) UV-vis spectral changes of 0.1 M glycine-HCl buffer at pH 3 and 25°C after NO gas bubbling into the solution. The initial spectrum is shown in blue, and the final spectrum at 120 min is shown in red. The characteristic tetrad of peaks at 347, 358, 371 and 386 nm indicates the generation of HNO_2 by disproportionation of NO in acidic condition as discussed in the main text. (b) The decay of the absorbance at 358 nm in the presence and absence of hemoCD-I. The result indicates that the disappearance of HNO_2 is not affected by hemoCD-I.

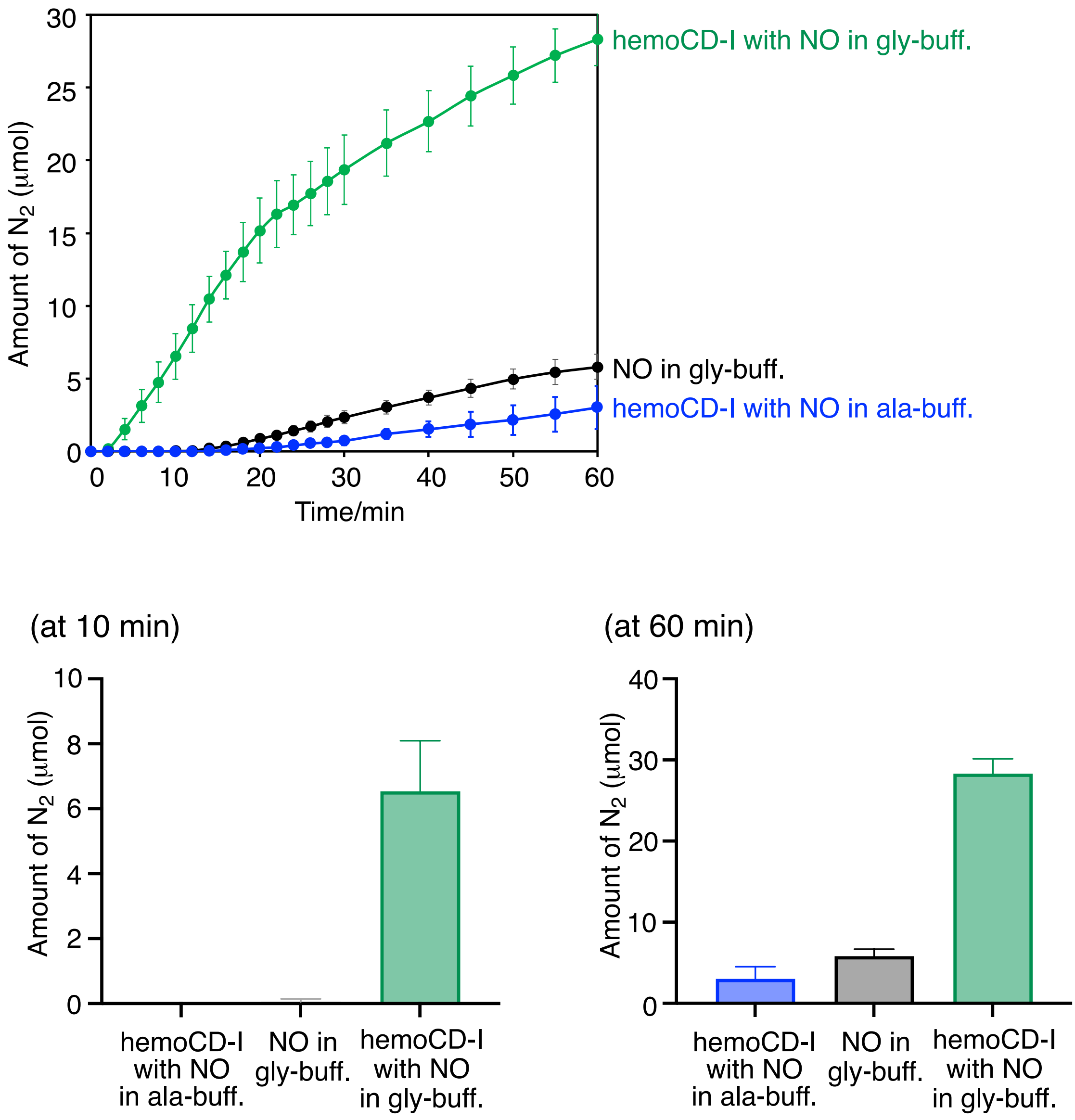

Figure S9. Quantification of generated N_2 gas from NO at pH 3 in the presence of hemoCD-I in 0.1 M Alanine-HCl buffer, and the comparison with hemoCD-I/glycine system. N_2 production of hemoCD-I in alanine buffer was not significant but almost same level to the blank glycine system.

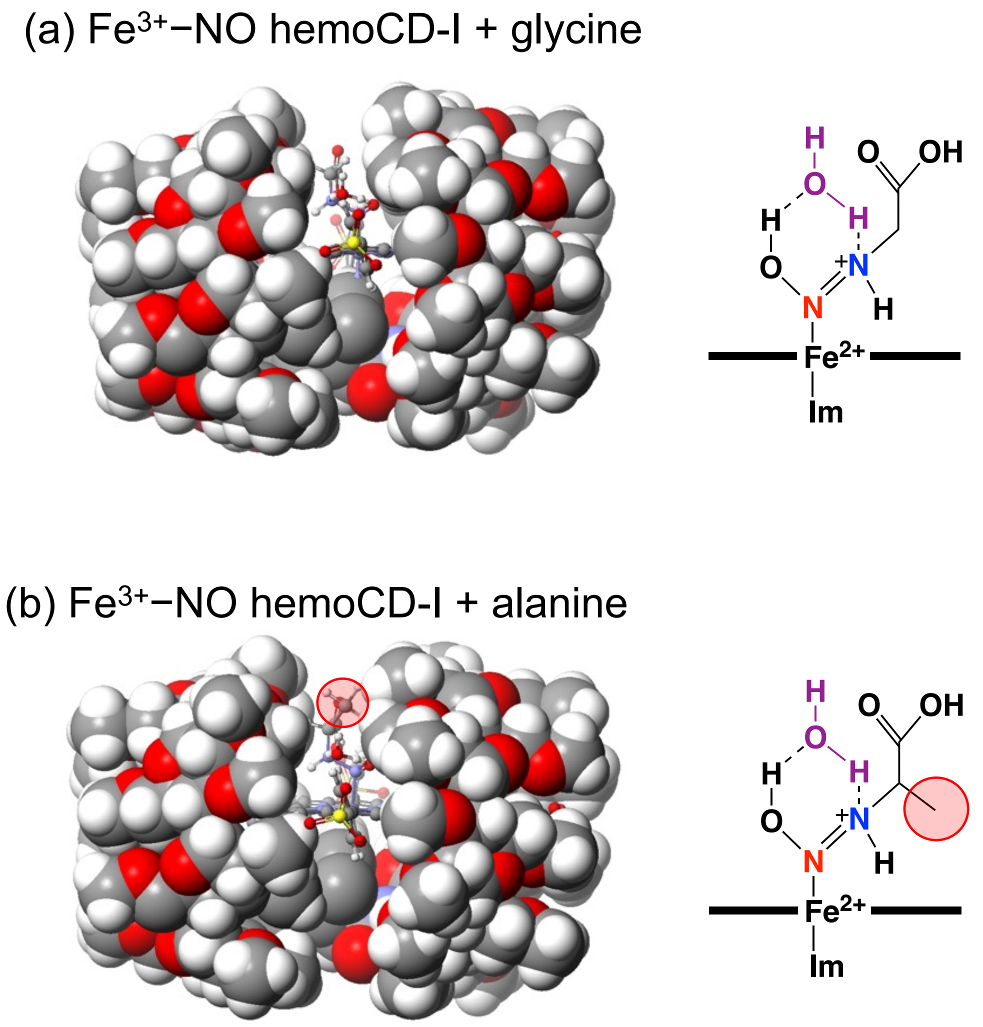

Figure S10. The molecular models of the proposed intermediates after the attack of (a) glycine and (b) alanine using CONFLEX/MM3 (extensive search) parameters in Scigress version 2.2.1 software program (Fujitsu). The CD moiety is shown in van der Waals sphere style, and the bound diazo compound and FeTPPS are shown in stick and ball style. A methyl group derived from alanine is indicated by a red circle which would cause the steric hindrance in the narrow cleft between two methylated cyclodextrin.
